# Tryptophan 32-mediated SOD1 aggregation is attenuated by pyrimidine-like compounds in living cells

**DOI:** 10.1038/s41598-018-32835-y

**Published:** 2018-10-22

**Authors:** Edward Pokrishevsky, Luke McAlary, Natalie E. Farrawell, Beibei Zhao, Mine Sher, Justin J. Yerbury, Neil R. Cashman

**Affiliations:** 1 Djavad Mowafaghian Centre for Brain Health, University of British Columbia, Vancouver, BC V6T 2B5 Canada; 2 Faculty of Science Medicine and Health, University of Wollongong, Wollongong, NSW 2522 Australia; 3 Illawarra Health and Medical Research Institute, University of Wollongong, Wollongong, NSW 2522 Australia

## Abstract

Over 160 mutations in superoxide dismutase 1 (SOD1) are associated with familial amyotrophic lateral sclerosis (fALS), where the main pathological feature is deposition of SOD1 into proteinaceous cytoplasmic inclusions. We previously showed that the tryptophan residue at position 32 (W32) mediates the prion-like propagation of SOD1 misfolding in cells, and that a W32S substitution blocks this phenomenon. Here, we used *in vitro* protein assays to demonstrate that a W32S substitution in SOD1-fALS mutants significantly diminishes their propensity to aggregate whilst paradoxically decreasing protein stability. We also show SOD1-W32S to be resistant to seeded aggregation, despite its high abundance of unfolded protein. A cell-based aggregation assay demonstrates that W32S substitution significantly mitigates inclusion formation. Furthermore, this assay reveals that W32 in SOD1 is necessary for the formation of a competent seed for aggregation under these experimental conditions. Following the observed importance of W32 for aggregation, we established that treatment of living cells with the W32-interacting 5-Fluorouridine (5-FUrd), and its FDA approved analogue 5-Fluorouracil (5-FU), substantially attenuate inclusion formation similarly to W32S substitution. Altogether, we highlight W32 as a significant contributor to SOD1 aggregation, and propose that 5-FUrd and 5-FU present promising lead drug candidates for the treatment of SOD1-associated ALS.

## Introduction

Amyotrophic lateral sclerosis (ALS) is a neurodegenerative disease characterised by the selective degeneration of upper and lower motor neurons leading to patient death typically within 2 to 4 years of onset. Approximately 90% of cases are sporadic (sALS), with remaining cases termed familial (fALS), as they are mostly linked to autosomal dominant mutations in one of several genes, including superoxide dismutase 1 (SOD1)^1^. A characteristic neuropathological feature of ALS is the formation of cytosolic protein inclusions in motor neurons and glia. The observed death of motor neurons in SOD1-associated ALS (SOD1-fALS) may be caused by a gain of cytotoxic function resulting from mutant SOD1 aggregation-induced dysregulation of the ubiquitin-proteasome system, mitochondrial dysfunction, disruption of cytoskeletal elements, sequestration of essential proteins, and potential generation of reactive nitrogen or oxygen species^2–4^. Although all mutations in SOD1 result in protein misfolding to different degrees^5,6^, the propensity of mutant SOD1 to aggregate may differ^7,8^, perhaps due to the existence of multiple conformational isoforms of pathological SOD1. In fact, prion-like “strains” of SOD1 aggregates can co-exist in experimental mouse models of ALS^9,10^, which have been found to transmit distinct molecular pathology^11,12^. The propagation of different strains of SOD1 aggregates suggests the existence of distinct “seeding species” possessing unique structural features.

The SOD1 sequence contains over 160 mutations (http://alsod.iop.kcl.ac.uk), all of which are associated with the ALS syndrome and pathological neuronal inclusion bodies. Although these mutations can have disparate effects upon the SOD1 structure by disrupting metal ion coordination, disulphide bond formation or reduction, and protein dimerization, they all engender SOD1 misfolding and subsequent aggregation^13–16^. Studies using ALS mouse models have demonstrated that overexpression of human wild-type SOD1 (hSOD1) can co-aggregate and accelerate disease of the hSOD1-G85R expressing mouse, but not that of mice expressing murine SOD1-G86R^17,18^. The lack of induced aggregation of mouse SOD1 by a hSOD1 seed has also been confirmed in cell culture^19^, even though human and mouse SOD1 have 83% sequence identity, with divergence mainly in β-strand III and loop II^19,20^. β-strand III contains the only tryptophan residue found within SOD1 at sequence position 32 (W32), which is solvent exposed in the native structure and is conserved across SOD1-fALS. In contrast to humans, mouse SOD1 contains no tryptophan residues, and position 32 is occupied by serine (S32). We have previously established that transmission of SOD1 misfolding can be significantly reduced by substituting W32 in hSOD1 by serine^19^ and other work determined that W32 potentiates mutant SOD1 aggregation and toxicity in a neuronal cell system^21^, further indicating the importance of this residue to pathology. More recently, 5-Fluorouridine, a chemotherapy agent, was shown to co-crystallize with W32 and other local residues on the surface of SOD1^22^, suggesting that this may be a potential target for attenuation of aggregation through blockade of Trp-Trp interactions.

Here, we investigated the effect of a W32S substitution on features related to SOD1 aggregation in isolated protein and cell culture models. We determined that recombinant SOD1 mutants harbouring an S32 substitution have decreased fibrillation propensities even though a higher proportion of the protein is found to be in an unfolded state if W32 is absent. Next, we observed that cell cultures expressing SOD1-S32 variants formed fewer inclusions at a slower rate than their W32-containing counterparts. In a cell-based seeded aggregation model, we found that S32 mutant SOD1 induces significantly less aggregation of SOD1 substrate, when compared to W32-containing counterparts. Finally, we determined that the small molecules 5-Fluorouridine, and the related FDA-approved, 5-Fluorouracil, were effective at attenuating the formation of SOD1 inclusions in a cell culture model.

## Results

### W32S substitution decreases both SOD1 stability and fibrillation propensity

**Figure 1:**
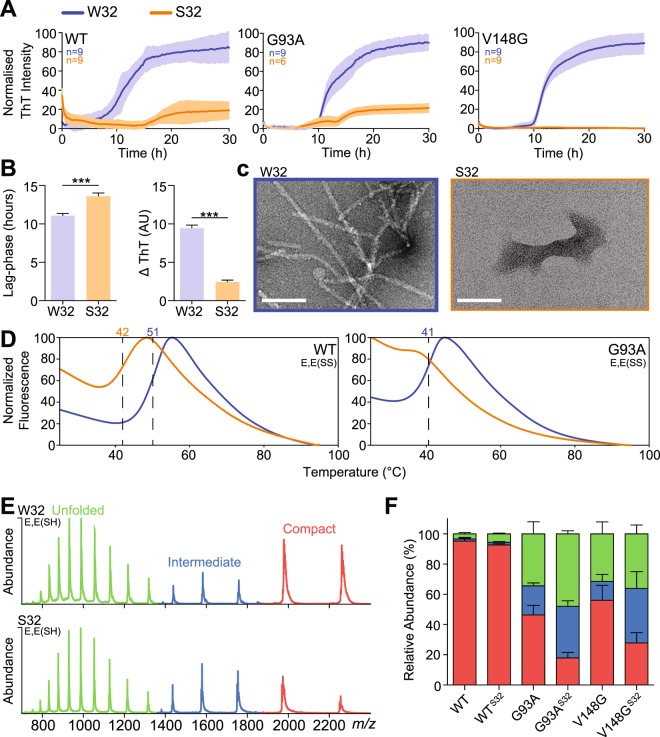
S32 substitution mitigates aggregate formation of purified SOD1 mutants and lowers their stability. (**A**) SOD1 aggregation was monitored by ThT fluorescence. W32S substitution decreased the ThT signal significantly for all variants (n = 9 wells across 3 assays). (**B**) Analysis of the aggregation kinetics of G93A-W32 and G93A-S32 where the lag-phase was significantly increased and the elongation rate was significantly decreased for G93A-S32. N values are the same as listed in the G93A plot in panel A, significance was determined by student’s t-test (***p < 0.01). (**C**) Electron micrographs of material from aggregation assays showing fibrils for G93A-W32 but small amorphous structures for G93A-S32. (**D**) DSF melt curves of E,E(SS) SOD1 variants showing the transition midpoint (Tm) for each mutant with a dotted line and corresponding temperature above (blue = W32, orange = S32). (**E**) Mass spectra of E,E(SH) G93A-W32 and G93A-S32 showing the charge state distribution where G93A-S32 has a greater abundance of protein populating unfolded (green) and intermediate (blue) conformations. (**F**) Analysis of mass spectra showing that a W32S mutation destabilises E,E(SH) for multiple variants. For panel F, error bars represent SD of at least 3 biological replicates with at least 2 technical replicates. Significance was determined using a one-way ANOVA with Tukey’s post-test (**p < 0.05; ***p < 0.01). Scale bar, 100 nm.

In order to determine the importance of W32 to SOD1 fibrillation, we generated recombinant SOD1 variants (wild-type, G93A, and V148G) containing an S32 substitution. First, we compared the *in vitro* fibrillation kinetics of W32 variants to that of their S32 counterparts using the fluorescent reporter of β-sheet formation, thioflavin T (ThT) in an agitating aggregation assay with reduced demetallated SOD1 (E,E(SH))^23^. The main kinetic parameters determined were the lag-phase of fibrillation, which is associated with the formation of detectable levels of β-sheet, and the elongation rate (measured as ∆ThT signal), which is associated with the rate at which β-sheet is formed. We observed that the S32 variant of each SOD1 mutant displayed significantly lower ThT binding responses compared to their W32 counterparts, consistent with decreased fibrillation propensities for the S32 variants (Fig. 1A). The only S32 variant to display typical sigmoidal fibrillation kinetics was G93A-S32, which showed a significantly increased lag-phase and decreased fibril elongation rate compared to G93A (~1.2 and ~4 fold, respectively; Fig. 1B), and a considerably decreased ThT signal at endpoint (~6.5 fold). Both WT and V148G followed sigmoidal kinetics in ThT fluorescence, but their S32 counterparts did not show an appreciable increase in ThT fluorescence. Transmission electron microscopy (TEM) revealed that G93A ThT binding was associated with fibril formation, whereas G93A-S32 TEM structures were sparsely distributed amorphous aggregates (Fig. 1C), ruling out the generation of ThT negative SOD1 fibrils as has been reported^24^.

In light of the substantial reduction in fibrillation propensity of the S32 variants, we were curious to determine the effect of W32S substitution on the stability of SOD1 as it has been reported to loosely correlate with SOD1 aggregation propensity and patient lifetime^25^. Therefore, we used differential scanning fluorimetry (DSF)^26^ to compare protein stability by measuring the average melting temperature of each variant. Importantly, apo-oxidized SOD1 (E,E(SS)) was used for this experiment due to the effect of metals to stabilize SOD1 to such a degree that accurate stability data cannot be determined using DSF, and the existence of differentially metallated SOD1 confounding analysis of data. We found that the E,E(SS) S32 variants showed high fluorescence intensity at the equilibration phase of the DSF experiments (Fig. 1D), suggesting that they were partially unfolded at ambient temperature. We were able to obtain measurable transition midpoints, from which T_m_’s are determined, for WT (51.1 ± 0.2 °C), WT-S32 (42.8 ± 0.1 °C), and G93A (41.0 ± 0.5 °C) (Fig. 1D). G93A-S32 was not measurable due to the absence of a transition midpoint. The T_m_ values obtained for E,E(SS) WT were close to the reported values of ~52 °C^27^. This instability was also examined orthogonally with limited proteolysis using proteinase K (PK). Incubation of apo-SOD1 forms with 1 and 10 µg/mL of PK resulted in the digestion of all apo-SOD1 variants, but not the holo (Cu,Zn(SS)) variants (Supplementary Fig. 2A). For both WT and G93A, a great proportion of the S32 form was degraded by PK at both concentrations, suggesting that W32S substitution facilitated the access of PK to the polypeptide backbone due to unfolding of the protein (Supplementary Fig. 2B). Together, these results suggest a crucial role of W32 in maintaining the structure and stability of SOD1 in the context of both WT and G93A variants, consistent with our observation from the above TEM and DSF analysis.

Following the determination that W32 was important for protein stability, we wanted to elucidate the effects of the W32S substitution on the conformation of the highly fibrillation-prone E,E(SH) SOD1 state^28,29^, which is thought to be the primary fibrillation precursor for SOD1 conformations^30^. We have previously used native mass spectrometry (MS) to obtain low-resolution assessment of SOD1 mutant conformations in this state^31^. Examination of the number of solvent exposed basic residues that give rise to the charge state distribution seen in mass spectra can delineate folded and unfolded states. Monomeric Cu,Zn(SS) SOD1 typically shows a bimodal charge state distribution with low charged +7 and +8 ions arising at low abundance comparative to the dimer (S1)^32^. Here, we find that the charge state distribution for E,E(SH) variants were broad, ranging from +7 to +21 for all mutants, indicating the exposure of a greater number of basic residues to solvent during ionisation (Fig. 1E), suggesting significant unfolding^31^. Although the charge state distributions between mutants were equal, S32 variants were found to possess higher abundances of charge-states from +9 to +21 relative to their W32 counterparts, suggesting a greater proportion of unfolded SOD1 after reduction and metal chelation (Figs 1E and S1). Quantification of the abundance of each charge state reveals that in each case the S32 variants have a significantly greater abundance of unfolded protein (Fig. 1F).

### Mutant SOD1 containing a W32S substitution is resistant to seeded aggregation *in vitro*

**Figure 2:**
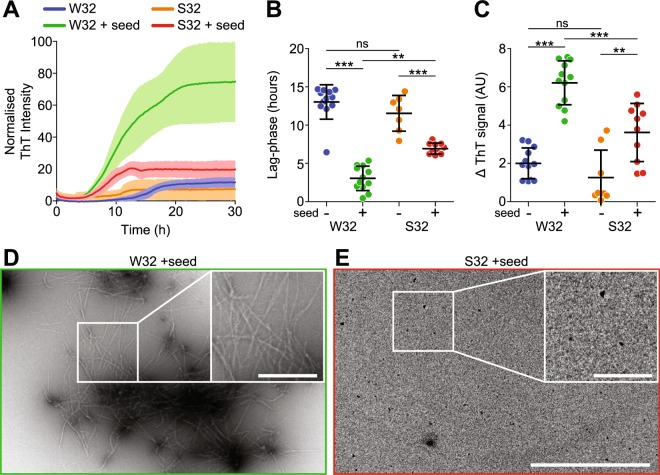
W32 mediated seeded SOD1 aggregation in a protein-only system. (**A**) Representative ThT binding assay of the seeded aggregation of G93A-W32 and G93A-S32 (colours correspond to labels in panel B). (**B**) Analysis of the lag-phase and (**C**) elongation rate from multiple ThT binding assays shows that G93A-S32 is resistant to seeding. Data points represent individual wells from 3 separate biological replicates. (**D**-**E**) Electron micrographs of aggregates produced from panel **‘A’** showing clear fibrils for G93A (**D**) and amorphous structures for G93A-S32 (**E**). Error bars represent SD of the mean for W32 (n = 12), W32 + seed (n = 12), S32 (n = 7), and S32 + seed (n = 10). See Supplementary Table 1 for more details. Significance was determined using a one-way ANOVA with Tukey’s post-test (ns = not significant; **p < 0.01; ***p < 0.001). Scale bar, 2 µm; insets, 0.5 µm.

Since S32 variants showed a paradoxically greater abundance of unfolded protein, but a significantly reduced fibrillation propensity compared to mutants possessing native W32, we hypothesised that the W32S substitution hinders the formation of a competent seed necessary for the genesis of β-sheet rich fibrils required for ThT fluorescence detection. To bypass the requirement for competent S32 seeds, we performed fibrillation assays of E,E(SH) G93A-S32 seeded by pre-formed G93A fibrils (Fig. 2A). We observed that even with G93A seeds that are highly competent to induce aggregation in G93A, the G93A-S32 substrate was resistant to fibrillation, displaying altered fibrillation kinetics with an increased lag-phase (~3 fold, Fig. 2B), reduced elongation rate (~4 fold, Fig. 2C), and lower ThT signal at endpoint (~5 fold) (Fig. 2A). Visual inspection of the end points of aggregation with TEM showed fibrils for seeded G93A (Fig. 2D and inset), and sparsely distributed small amorphous aggregates for seeded G93A-S32 (Fig. 2E and inset). Within the context of the microscopic events known about ThT-based assays of fibrillation^33^, specifically fibril elongation, the observed lack of ThT fluorescence signal for G93A-S32 in our assays is indicative of it being a poor substrate for preformed SOD1 fibrils.

### W32S substitution significantly lowers SOD1 inclusion formation in living cells

Having determined that W32S substitution significantly decreased the aggregation propensity of SOD1 in isolated protein systems, we were encouraged to assess its effect in cell-based models of SOD1 aggregation, where the environment is more complex and cellular processes play a role in inclusion formation. Therefore, we examined the effects of the W32S substitution on the inclusion formation of multiple SOD1 mutants in motor-neuron like NSC-34 cells using a GFP-tagged SOD1 transient transfection system. Counting the number of cells containing inclusions showed that S32 variants formed fewer inclusions than their W32 counterparts (Supplementary Fig. 3A), similar to its effect in our isolated protein assays. Time-lapse microscopy quantifying the number of GFP-positive cells post-transfection revealed that the W32S substitution in all cases engendered reduced cell death comparative to their W32 counterparts (Supplementary Fig. 3B). Co-transfections of G85R-W32-GFP or G85R-S32-GFP with fluorescent reporters of ubiquitin proteasome dysfunction (tdTomato-CL1) or endoplasmic reticulum stress (XBP1-tdTomato) both showed significantly lowered fluorescence signal for G85R-S32 in comparison with G85R-W32 (Supplementary Fig. 2F), suggesting that W32 is important in aggregation and toxicity pathways that are shared amongst SOD1 mutants. Since S32 substitution decreased the inclusion formation of multiple mutants including G93A and G85R, and as the obligate misfolded monomer G85R is a promiscuous acceptor of seed-induced SOD1 aggregation^11,34^, we focused our quantitative time-lapse cell-based aggregation studies on the pathogenic G85R mutant.

**Figure 3:**
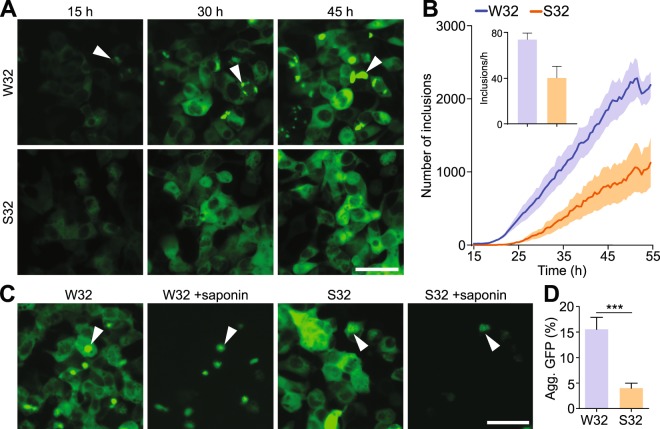
S32 substitution decreases the aggregation propensities of SOD1 mutants in living cells. (**A**) Time-lapse microscopy of G85R-W32-GFP and G85R-S32-GFP expressing HEK293FT cells shows bright puncta for G85R-W32-GFP but not G85R-W32S-GFP. (**B**) Quantitation of time-lapse microscopy reveals G85R-S32-GFP (orange) aggregates to a lesser extent compared to G85R (blue). Inset is the rate of inclusions detected per hour. (**C**) Cells transfected with either G85R-W32-GFP or G85R-S32-GFP were treated with saponin 48 h post-transfection and imaged pre- and post-treatment. (**D**) Analysis of saponin insoluble GFP signal shows a significantly higher amount of aggregated GFP for G85R-W32-GFP following saponin treatment. Error bars represent SEM of at least 3 biological replicates. Significance was determined using students t-test (***p < 0.001). Scale bar, 50 µm.

We first compared the inclusion formation across time between G85R-W32-GFP and G85R-S32-GFP in living cells using time-lapse microscopy. Visual inspection of the cells demonstrated that G85R-GFP forms more inclusions relative to G85R-S32-GFP throughout the time-course of the assay (Fig. 3A, Movie 1). Quantitative analysis of the images^34^ demonstrated that during the early inclusion formation phase which displays linear kinetics (10 to 33 h), G85R-W32-GFP formed inclusions at nearly double the rate of the G85R-S32-GFP variant (Fig. 3B and inset). In order to confirm that the soluble cytosolic fluorescent signal of SOD1-GFP does not obscure inclusions, we treated the cells using the detergent saponin at the assay end-point, allowing the soluble SOD1-GFP protein to diffuse out of the cells while retaining the inclusions (Fig. 3C). Quantification of the remaining GFP positive inclusions following permeabilization, demonstrated a statistically significant 3-fold reduction in the numbers of inclusions in G85R-S32-GFP vs G85R-W32-GFP expressing cells (Fig. 3D).

### Seeded aggregation of SOD1 in cells is mediated by tryptophan 32

**Figure 4:**
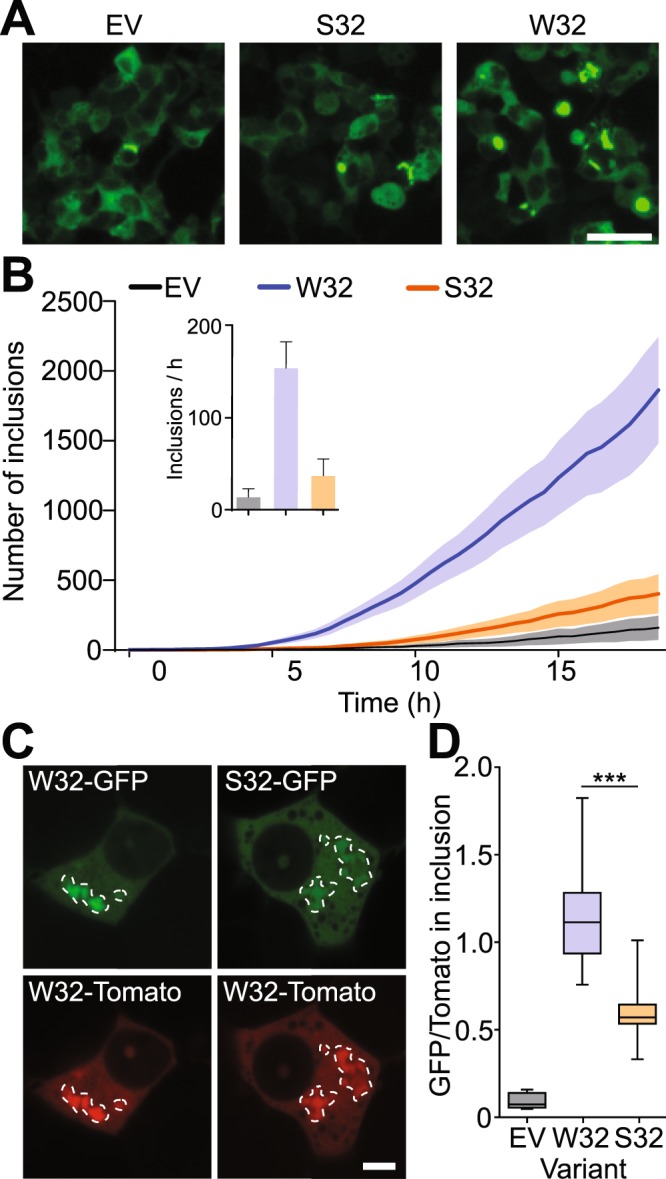
SOD1-S32 is resistant to seeded aggregation in cells. (**A**) Images from time-lapse microscopy of seeded aggregation. (**B**) Algorithm counting of aggregates in living cells from 24 to 48 h post co-transfection with G85R-GFP reporter protein and indicated non-tagged construct. Images were acquired every 30 minutes. Inset indicates the rate of inclusion formation per hour during the inclusion growth phase. (**C**) Co-transfection of G85R-W32-GFP and G85R-W32S-GFP with G85R-tdTomato shows clear intense GFP fluorescence co-localized with tdTomato fluorescence for G85R-W32-GFP but not G85R-W32S-GFP. (**D**) Analysis of the GFP fluorescence intensity that overlaps with the G85R-TdTomato inclusions shows that G85R-W32S-GFP intensity is significantly lower than that of G85R-W32-GFP. Error bars on bar charts and line plots indicate SEM of at least 3 biological replicates. For panel D box-whisker plots, centre line-mean; box limits-upper and lower quartile; whiskers-0 and 100% range. Significance was determined via students t-test (***p < 0.001). Scale bar, 50 µm (**A**); 10 µm (**C**).

Since purified protein assays showed that W32S substitution lowers the propensity of SOD1 to aggregate in the presence of competent SOD1 seed, we next explored this phenomenon in living cells using a co-transfection system where one vector encoded a fluorescent reporter of inclusion formation and the other induced inclusion formation (untagged mutant SOD1 or control). First, we performed time-lapse microscopy on HEK293FT cells co-transfected with G85R-W32-GFP reporter protein and non-tagged G85R-W32, G85R-S32, or empty vector control inducers. Transient transfection of the reporter protein was titrated to minimize spontaneous aggregation^34^. We found that G85R-S32 inducer is not an effective promoter of G85R-W32-GFP aggregation (Fig. 4A,B, Movie 2), and estimated a 5-fold decrease in the rate of inclusion formation during the rapid inclusion growth phase (from 8 to 17 h), when compared to untagged G85R inducer (Fig. 4B and inset). The inability of G85R-S32 to induce aggregation of the reporter protein is especially important in light of the fact that both G85R-W32 and G85R-S32 express equally well in HEK293FT cells, and both appear to be at least partially misfolded in the cytosolic environment, as determined by the SOD1 misfolding specific antibody 3H1^19,35^ (Supplementary Fig. 2E).

Having established that seeded aggregation of G85R-W32-GFP by non-tagged G85R-S32 is significantly reduced compared to G85R, we next wanted to examine to what extent S32 variants were co-aggregating with W32 mutants. To this end, G85R-tdTomato was co-expressed with G85R-W32-GFP or G85R-S32-GFP in NSC-34 cells by transfecting them at a 1:1 ratio. The fluorescent signal of GFP and tdTomato in individual inclusions was measured and normalized to the diffuse signal of soluble fusion protein within the cell body. We found that G85R-GFP and G85R-S32-GFP fluorescent signal in G85R-tdTomato inclusions was greater than the diffuse cellular signal (Fig. 4C), indicating co-aggregation of the fluorescent constructs above the background of soluble protein. However, analysis comparing the ratio of GFP to tdTomato fluorescent signal in the inclusions showed a significant 2-fold decrease of G85R-S32-GFP signal in the inclusions (Fig. 4D), consistent with its resistance to aggregation observed previously in the seeding of recombinant proteins (Fig. 2). This phenomenon was not restricted to G85R as we observed a similar 2-fold decrease using A4V, G93A, and V148G in the same assay (Supplementary Fig. 2C,D).

### Inhibition of tryptophan-mediated seeded aggregation of SOD1 using small molecule drugs

**Figure 5:**
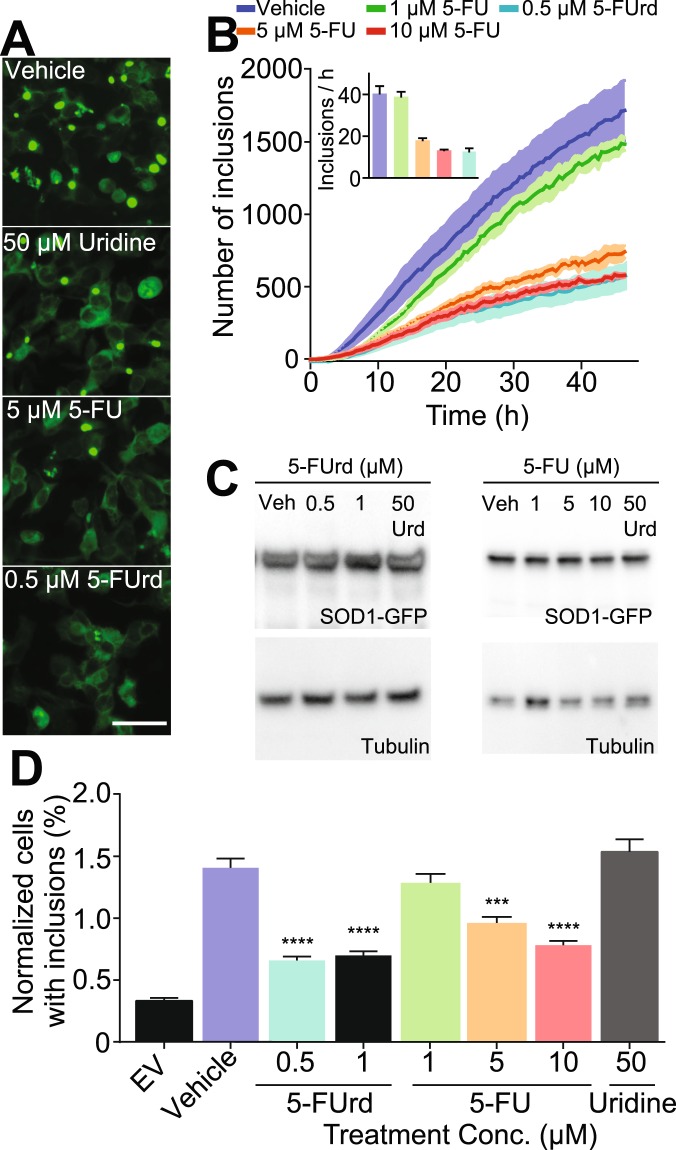
Small molecules targeting W32 can effectively inhibit inclusion formation in living cells. (**A**) Images from 48 h post-transfection of chemically treated cells. (**B**) Quantification of time-lapse live-cell microscopy of seeded aggregation showing that 5-FUrd and 5-FU can effectively decrease SOD1 inclusion formation. (**C**) Immunoblots of chemically treated cells showing similar levels of SOD1 expression regardless of treatment. Uncropped blots can be found in the supplementary information (Supplementary Figs 6–9). (**D**) Flow cytometry of saponin treated cells showing a reduced number of inclusion containing cells in both 5-FUrd and 5-FU treatment groups. For each experiment above, error bars represent SEM of at least 3 biological replicates. Statistical significance was determined using one-way ANOVA and Dunnett’s test for multiple comparisons (***p < 0.001; ****p < 0.0001). Scale bar, 50 µm.

Having established that a W32S substitution can significantly decrease the aggregation propensity of SOD1 mutants *in vitro* and in cells, we were curious if small compounds that have previously been shown to interact with W32 could achieve similar results. The pyrimidine derivative 5-Fluorouridine (5-FUrd) has been shown to interact with SOD1 at W32 by x-ray crystallography^22^, therefore, we set out to determine if 5-FUrd and related compounds can block SOD1 seeded aggregation in our previously described inclusion inducing cell model. Immunofluorescence of transfected cells showed that as little as 0.5 µM 5-FUrd, and 5 µM of a related FDA-approved chemotherapeutic 5-Fluorouracil (5-FU), significantly lowered SOD1 seeded aggregation in our live-cell assays (Fig. 5A). We did not test these drugs on G85R-S32-induced aggregation of the reporter protein as we previously demonstrated (Fig. 4A,B), that in stark contrast to W32, G85R-S32 does not induce a significant increase in aggregation of our reporter protein.

Time-lapse microscopy showed that 0.5 and 1 µM 5-FUrd are equally effective at reducing inclusion formation (Movie 3, Fig. 5B and inset), with the rate of inclusion formation decreasing by nearly 3-fold with drug treatment. Furthermore, 5-FU lowered the rate of inclusion formation of G85R-W32 in a dose dependent manner, with a statistically significant 3-fold decrease in response to a 5 or 10 µM treatment (Fig. 5B). These results were confirmed using flow cytometry of saponin-permeabilized cells, demonstrating that 0.5 and 1 µM 5-FUrd, as well as 5 and 10 µM 5-FU, significantly lower the seeded aggregation of SOD1 (Fig. 5D). Although a concentration-dependent reduction in aggregation was also detected with 1 µM 5-FU, statistical significance was not achieved (Fig. 5D). Treatment of cells with the non-fluorinated pyrimidine base uridine did not lower inclusion formation at 50 µM (Fig. 5).

As 5-FUrd and 5-FU are known to reduce RNA transcription, we confirmed by immunoblotting that the expression levels of G85R-W32-GFP are maintained in the presence of these drugs (Fig. 5C and Supplementary Figs 6–9). In 5-FU cancer chemotherapy, uridine has been employed as a rescue agent. Here, we confirmed that the combination of 5-FUrd or 5-FU and uridine is equally effective at lowering seeded aggregation of SOD1 (Supplementary Fig. 2G).

## Discussion

Mounting evidence has demonstrated that proteins implicated in neurodegenerative diseases, such as Alzheimer’s (amyloid-β, tau), Parkinson’s (α-synuclein) and ALS (SOD1 and TDP-43), feature pathological propagation of protein aggregation, which is thought to be due to the recruitment of soluble proteins into aggregates through a prion-like seeding mechanism^36^. Differences in pathological propagation may be a consequence of conformational heterogeneity and molecular-level polymorphism in the protein^37^. Previously, several segments throughout the SOD1 sequence (14–21, 30–38, 101–107, and 147–153) have been shown to be fibrillogenic^38^. Importantly, segment 30–38 contains W32, and as a peptide has been shown to form structured oligomers^39^, displaying toxicity to motor neurons^40^. The notion of SOD1 aggregate strains has been demonstrated using a binary epitope mapping strategy in SOD1-D90A mice, in which two structurally distinct aggregate strains were identified^9^. Neither strains co-aggregate with endogenous mouse SOD1, which has substantial sequence divergence from hSOD1 sequence only in this segment^19^. Furthermore, studies of the folding trajectories of E,E(SH) SOD1 have suggested that SOD1 folds through a transition state where β-strands I-IV form a stable β-sheet core^29,41–43^. This is important for aggregation onset, considering that nascent SOD1 monomers require a relatively long time to fold^44^. If a transition state is populated for longer periods, due to mutation, there is a possibility of non-native interactions leading to oligomerisation and aggregation. When undergoing fibrillogenesis *in vitro*, SOD1 mutants are suggested to present highly stochastic fibrillation kinetics, resulting in a mixture of amorphous and fibrillar aggregates^45^, which compete for soluble monomeric substrate. In light of this work, there is potential for specific states to be populated by mutant SOD1 that shifts towards fibrillar or amorphous pathways, and that in our assay conditions W32S substitution has tipped the scale towards amorphous aggregation. However, our results argue against widespread amorphous aggregation as these aggregates were only sparsely observed.

Interestingly, SOD1-fALS patients with a G37R mutation, which is contained within segment _30_KVWGSIKGL_38_, present longer disease durations compared to other SOD1-fALS patients^25^. This may be a consequence of G37R aggregating as a less toxic strain due to blocking formation or changing the structure of a β-strand III dependent aggregate. Furthermore, the peptide 30–38 has been demonstrated to be highly fibrillogenic unless a G37R mutation is present^38^. Indeed, transgenic mice expressing sub-disease levels of hSOD1-G85R-YFP, develop SOD1 aggregation and paralysis only when injected with spinal cord homogenate from G93A, but not G37R, transgenic mice^11^. Within this context, our data argue that the propensity of SOD1 to aggregate depends on the region encompassing β-strand III, and that W32 within this motif is a key participant in this process. This notion is further supported by the inability of recombinant reduced apo-G37R to be seeded by G93A fibrils *in vitro*^31^, even though G37R is mostly unfolded in this state and presents a high aggregation propensity in cells. Furthermore, it has been suggested that the aggregation prone disease conformation of SOD1 is a highly unfolded state^30^, however, here S32 variants present lower melting temperatures and greater abundance of protein in an unfolded conformation, but paradoxically lower aggregation propensities. This observation is interesting within the context of current understanding pointing towards increased unfolding leading towards increased aggregation, and suggests that this might not always be the case.

Although our data demonstrate that the W32S substitution plays a significant role in aggregation, it did not completely block aggregation in our assays. Previous work has suggested that oxidation of W32 can lead to the formation of covalently crosslinked non-native SOD1 dimers which could be a cytotoxic species or seed for aggregation^21,46,47^. We did not observe dimer signal in immunoblots of G85R-W32 transfected cells (Supplementary Figs 6–8), however, we cannot rule out that any W32-crosslinked SOD1 exists at levels undetectable in our assays. Contrary to this point, SOD1-S32 was not completely immune to aggregation in our protein and cell studies. This suggests that aggregation may be partially mediated through other aggregation prone segments in addition to W32^38^. Indeed, SOD1 in other species can also aggregate without tryptophan; for example, despite dogs and mice lacking W32, and having major sequence divergence at sequence segment 28–38 (canine = 64% identity, mouse = 55% identity) (Supplementary Fig. 10), dogs can develop canine degenerative myelopathy with SOD1 mutations^48^, while transgenic mice carrying a G86R mutation in murine SOD1 develop disease that features motor neuron degeneration and SOD1 positive inclusions^49,50^. Altogether, these data suggest that assembly of SOD1 into aggregates can proceed through different regions of the protein, but that a consensus aggregation pathway utilizes tryptophan. Our live-cell microscopy data also suggest that tryptophan-mediated aggregation of SOD1 is kinetically advantaged over aggregation that does not utilize tryptophan, reminiscent of kinetic factors operant in strain selection of infectious prions *in vivo*^51^.

The significance of the W32 residues involvement in the seeded aggregation of SOD1 goes beyond basic biology and mechanistic discovery – it suggests new therapeutic avenues. We determined that 0.5 µM 5-FUrd lowers the aggregation of SOD1 to levels comparable to that of controls. Original studies using 5-FUrd suggested that its mechanism of action is the stabilization of SOD1 dimer^52,53^. However, a co-crystallization study demonstrated that 5-FUrd interacts non-covalently with W32, mediated by the fluorouracil moiety^22^. We also find 5-FU, a pyrimidine drug that can effectively penetrate the blood-brain-barrier^54,55^, to attenuate SOD1 aggregation in cells. Owing to their disturbance of RNA/DNA metabolism, both 5-FUrd and 5-FU cause toxicity, which can be mitigated by co-administration with uridine^56,57^, a combination that we show here to be effective in blocking seeded aggregation of SOD1 in cells. Given the apparent role that W32 plays in the intermolecular induction of SOD1 aggregation, we propose that inhibition of W32-mediated aggregation using low dosages of 5-FUrd, 5-FU, or structurally similar compounds, should be explored as potential drugs against SOD1-fALS.

## Conclusion

A subset of familial ALS cases are associated with mutations in SOD1. A region encompassing the sole tryptophan at residue position 32 (W32) is important for pathological aggregation. We show that W32 governs the aggregation process of SOD1 mutants and that small molecules known to bind around W32 can mitigate this process in cells. We present two chemotherapy agents, 5-Fluorouridine and 5-Fluorouracil, as lead candidates for treatments directed against SOD1-associated ALS. This work also establishes a robust assay for the screening of compounds aiming to reduce SOD1 aggregation.

## Materials and Methods

### Thioflavin-T Binding Assays

Unseeded protein aggregation assays were carried out as described previously^31^. Briefly, purified recombinant SOD1 was diluted to a concentration of 60 µM monomer in the presence of 20 mM DTT (30 mM for SOD1-WT), 5 mM EDTA, and 10 µM Thioflavin T in 1 × PBS (pH 7.4). The aggregation mix was plated (final volume per well = 50 µL) into clear flat-bottomed 384-well plates (Greiner) and incubated at 37 °C for 30 min before being covered by a clear adhesive film. A POLARstar Omega plate-reader (BMG Labtech, Germany) was used to measure ThT fluorescence which was excited at 440 nm and emission was read at 490 nm using the bottom optic. Aggregation was induced with double-orbital shaking at 300 rpm for 330 s at the start of a 900 s cycle, with a constant temperature of 37 °C. Seeded protein aggregation assays were performed similar to above with the exception that only 10 mM DTT and 1 mM EDTA were used and seed (from a previous assay) constituted 0.3% (w/w) of total protein amount in each assay. Lower concentrations of DTT and EDTA were used to minimise aggregation of unseeded samples. Boltzmann sigmoidal relationships were fit using GraphPad Prism v5.0 and analysis of aggregation kinetics was carried out as previously described^58^. Since determination of the lag-phase of fibrillation and fibril elongation rate requires an acceptable fit of a Boltzmann sigmoid function to ThT binding data, cases where there was no sigmoidal response were not included in the analysis (see Supplementary Table 1).

### Native Mass Spectrometry

Purified SOD1 was destabilized via incubation in 20 mM DTT 5 mM EDTA in 1 × PBS (pH 7.4) at 37 °C for 2 h, following which samples were buffer exchanged into 200 mM ammonium acetate using gel-filtration chromatography (Superdex 75 10/300 GL, GE USA). Fractions off the column were immediately placed on ice, pooled, and diluted to a final concentration of 10 µM SOD1 monomer (measured via BCA assay). Mass spectrometry analysis was performed using a SYNAPT G1 HDMS (Waters, UK) with parameters set according to previous work^32^. Briefly, SOD1 samples at 10 μM in 200 mM NH_4_OAc were loaded into gold-coated borosilicate capillaries (made in-house) and subjected to nano-electrospray ionization. Results are representative of 3 separate unfolding experiments. All spectra were externally calibrated using 10 mg/ml caesium-iodide in 50% n-propanol, and were processed using Masslynx 4.1. For determination of the abundances of the observed conformations the area under the peak for each charge state was determined and the values plotted as a function of charge state. Following this, MATLAB R2014b (Version 8.4) was used to fit Gaussians to the plots and area under the peak was determined. Instrument parameters can be found in Appendix A.

### Fluorescent Imaging

Time-lapse live-cell imaging was carried out on HEK293FT cells in 8-well microscope chambers 24 h post-transfection under humidified conditions with 5% CO_2_ at 37 °C. Small molecules (5-FUrd and 5-FU), if indicated, were added 6 h post-transfection to allow for proper protein expression. Images were acquired at 1024 × 1024 pixel resolution (3 × 3 grid – stitched post-acquisition) were acquired every 30 min using an A-Plan 10× objective 0.25NA mounted on an inverted Axio Observer Z1 microscope (Carl Zeiss AG, Germany) equipped with an AxioCam HighRes camera (Carl Zeiss AG, Germany) and a motorized stage. Live-cell imaging started 20–24 h post transfection to allow minimal amount of reporter protein expression.

For antibody staining, cells were plated onto glass coverslips prior to transfection with the indicated construct as described above and collected 48 h post-transfection. Cells were fixed using 4% paraformaldehyde and stained with misfolding specific antibody 3H1, and pan-SOD1 antibody SOD100 (Enzo Life Sciences, USA) as previously described^20^.

### Image Analysis

To determine the ratio of GFP to tdTomato that formed inclusions in co-transfected cells, images were converted to 8-bit format and the area within a cell that was composed of aggregated SOD1 was determined by setting a ‘max entropy’ threshold in the tdTomato channel, followed by then applying this selected area to the green channel and measuring the pixel area (*A*_*P*_) and mean pixel intensity (*I*_*P*_) of both channels. The *A*_*P*_ and *I*_*P*_ of the diffuse fluorescent protein was determined in the tdTomato channel, and applied to the GFP channel, by setting the maximum threshold to the value of the minimum threshold determined for the aggregated fluorescent protein, followed by setting the minimum threshold to a value that selected the cell cytoplasm. Using *A*_*P*_ and *I*_*P*_ values for both diffuse and aggregated protein, in both the GFP and tdTomato channels, we determined the ratio of GFP to tdTomato signal that was forming inclusions per cell using the method described in the appendices.

### Flow Cytometry

HEK293FT cells were co-transfected with inducer and reporter proteins for 6 h prior to addition of the small molecules. 40–48 h later, the cells were washed 2 times with cold PBS and permeabilized using ice-cold 1 × PBS supplemented with 0.03% saponin (w/v) for 10 min on ice in order to allow diffusion of soluble intracellular GFP out of the cells in order to identify those cells with fluorescent inclusions using flow cytometry. Cells were then collected in a microcentrifuge tube, washed once in PBS and stored on ice until analysis on LSRII (BD Biosciences, USA). Analysis of living cells was performed using FlowJo. We selected cells positive to GFP inclusions by setting the gate in such way that it captures the most events in samples without drugs, but few events in cells co-transfected with reporter protein and empty vector. These settings were kept consistent for the entire data set, which were normalized to control across biological repeats.

## Electronic supplementary material

Movie S1

Movie S2

Movie S3

Supplementary Information

## Data Availability

All materials and data are available upon reasonable request to the corresponding authors.
