# Supplementary material for: Tryptophan 32-mediated SOD1 aggregation is attenuated by pyrimidine-like compounds in living cells
Source: Sci Rep. 2018 Oct 22;8:15590. doi: 10.1038/s41598-018-32835-y (PMC6197196; doi:10.1038/s41598-018-32835-y)
Supplement: Supplementary file 4 — Supplementary Information [file 41598_2018_32835_MOESM4_ESM.docx]

**Supplementary Information**

**Tryptophan32-mediated SOD1 aggregation is attenuated by pyrimidine-like compounds in living cells**

Edward Pokrishevsky^a1^, Luke McAlary*^b,c1^, Natalie E. Farrawell^b,c^, Beibei Zhao^a^, Mine Sher^a^_,_ Justin J. Yerbury^b,c2^, and Neil R. Cashman^a2^

^1^E.P and L.M contributed equally to this work

^a^Djavad Mowafaghian Centre for Brain Health, University of British Columbia, Vancouver, BC V6T 2B5, Canada

^b^Faculty of Science Medicine and Health, University of Wollongong, Wollongong, NSW 2522, Australia

^c^Illawarra Health and Medical Research Institute, University of Wollongong, Wollongong, NSW 2522, Australia

**^2^Corresponding Authors**

Neil R. Cashman; +1-604-822-2135, [neil.cashman@vch.ca](mailto:neil.cashman@vch.ca)

Justin J. Yerbury; +61-2-42-981-534, [jyerbury@uow.edu.au](mailto:jyerbury@uow.edu.au)

**Supporting Figures**


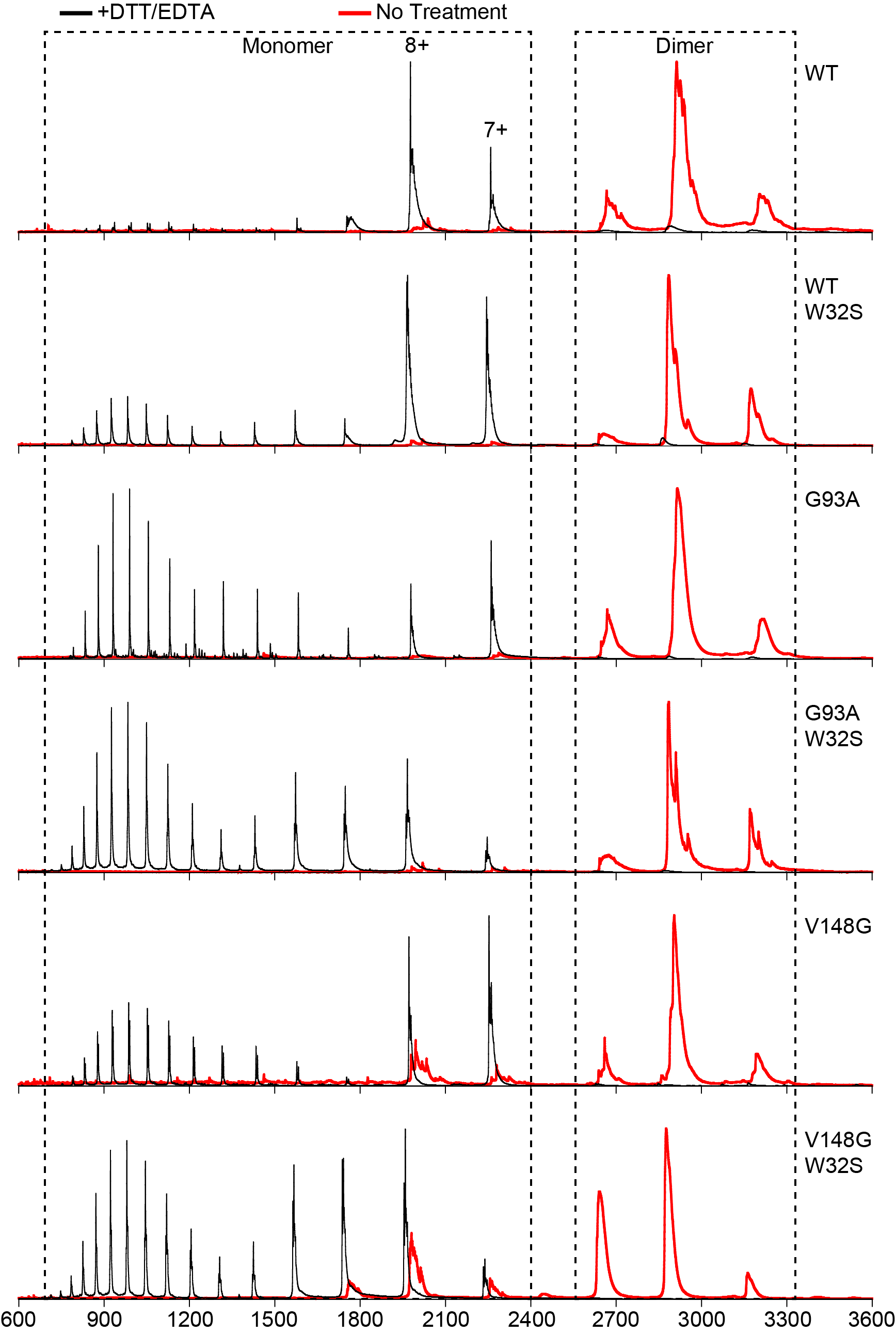


**Supplementary Figure 1.** Mass spectra of all SOD1 variants with no treatment (red traces) and DTT/EDTA treated (black traces) overlaid for each variant.

**Supplementary Table 1. Thioflavin T fibrillation assay data description.**

| Experiment | Total n (wells) | Sigmoidal response (fit above r^2^ = 0.8) | Replicates with sigmoidal response (%) |
| --- | --- | --- | --- |
| Figure 1 | | | |
| WT | 9 | 9 | 100 |
| WT-W32S | 9 | 0 | 0 |
| G93A | 9 | 9 | 100 |
| G93A-W32S | 9 | 6 | 67 |
| V148G | 9 | 9 | 100 |
| V148G-W32S | 9 | 0 | 0 |
| Figure 2 | | | |
| G93A | 12 | 12 | 100 |
| G93A + seed | 12 | 12 | 100 |
| G93A-W32S | 12 | 7 | 58 |
| G93A-W32S + seed | 12 | 10 | 83 |


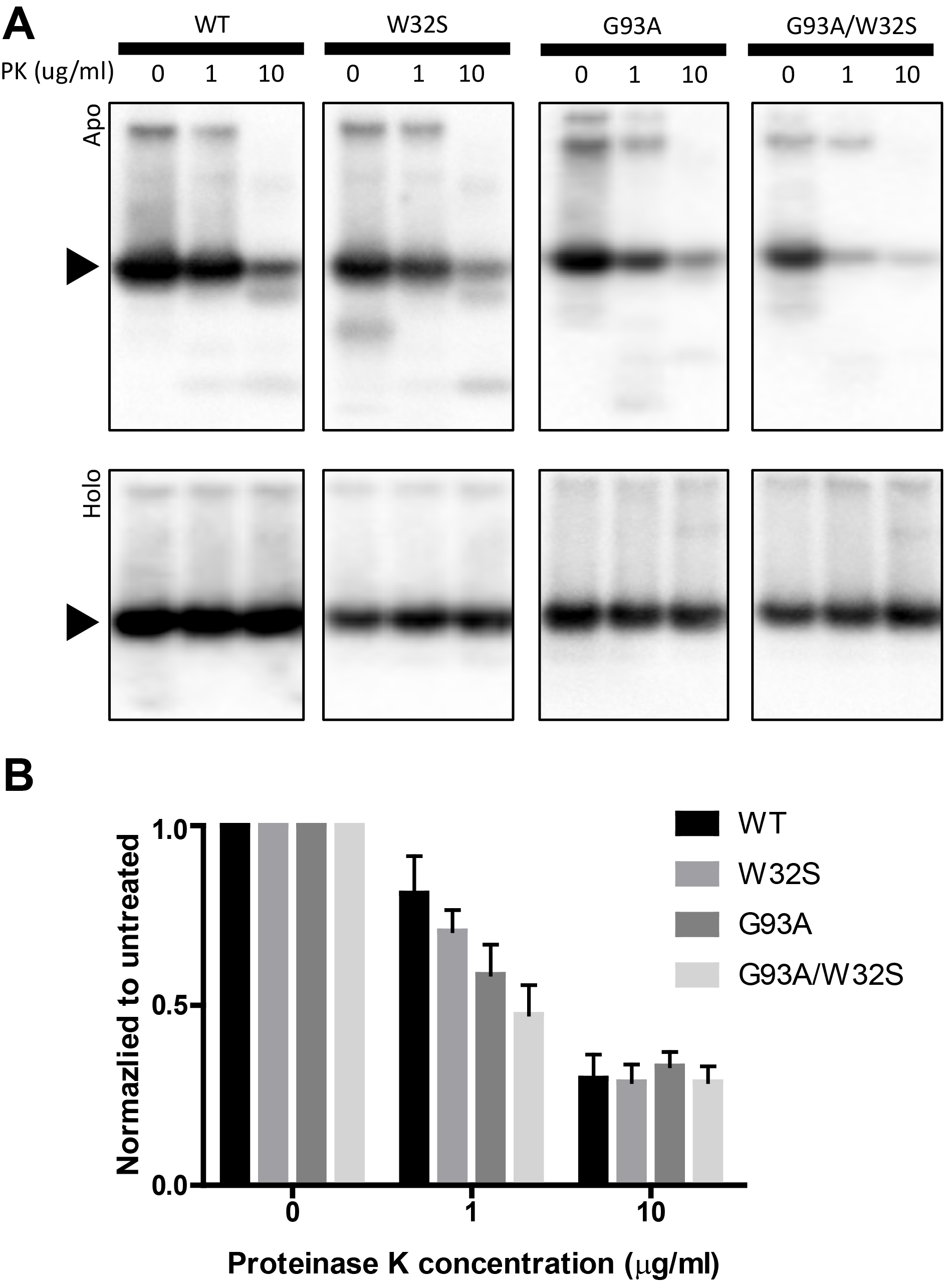


**Supplementary Figure 2. (A)** Immunoblots of proteinase K digested apo- and holo-SOD1. **(B)** Quantification of immunoblots (n=3).


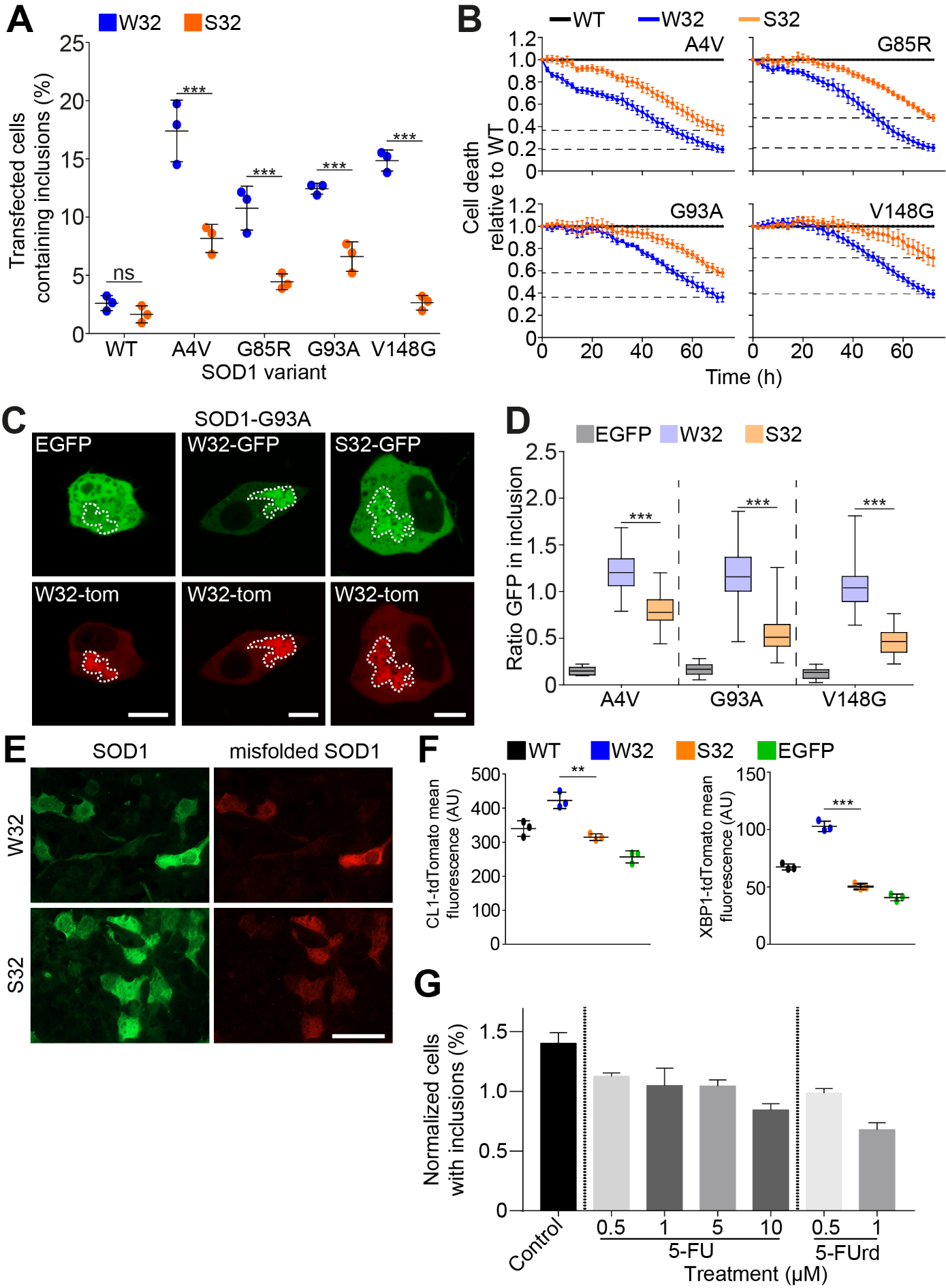


**Supplementary Figure 3. (A)** Percentage of NSC34 cells containing inclusions 48 hr post transfection. Error bars represent SD of 3 biological replicates. Significance was determined using one-way ANOVA with Bonferroni’s post-test (*ns =* not significant; *** *p* < 0.001) **(B)** Toxicity of the expression of SOD1 W32 and S32 variants in NSC34 cells normalised to SOD1-WT expression. Dotted lines indicate the proportion of cells alive at assay end point. **(C)** Representative images of NSC34 cells co-transfected with EGFP, W32-EGFP, or S32-EGFP, and W32-Tomato vectors. White outlines indicate ROI’s of W32-tdTomato inclusions. Scale bars are 10 µm. **(D)** Quantification of the relative EGFP fluorescence signal in ROI’s defined by W32-Tomato inclusions. Error bars represent minimum and maximum values from at least 3 biological replicates with at least 20 technical replicates each. Significance was determined using one-way ANOVA with Bonferroni’s post-test (*** *p* < 0.001). **(E)** Misfolded SOD1 is detected with monoclonal antibody 3H1 (red), and total SOD1 detected with SOD100 in HEK293FT cells transfected with S32 or W32 SOD1-G85R. Scale bar is 50 µm. **(F)** Flow cytometry analysis of cell co-transfected with SOD1 constructs and fluorescent stress reporters (Proteasomal Stress – CL1-tdTomato, ER Stress – XBP1-tdTomato) where increased fluorescence indicates increased stress. Error bars represent SD of 3 biological replicates. Significance was determined with a student’s t-test between W32 and S32 SOD1 (** *p* < 0.01, *** *p* < 0.001). **(G)** Addition of 50 µM Uridine with either 5-FU or 5-FUrd lowers the number of inclusions in treated cells. Flow cytometric measurement of the percentage of cells containing inclusions following saponin treatment.


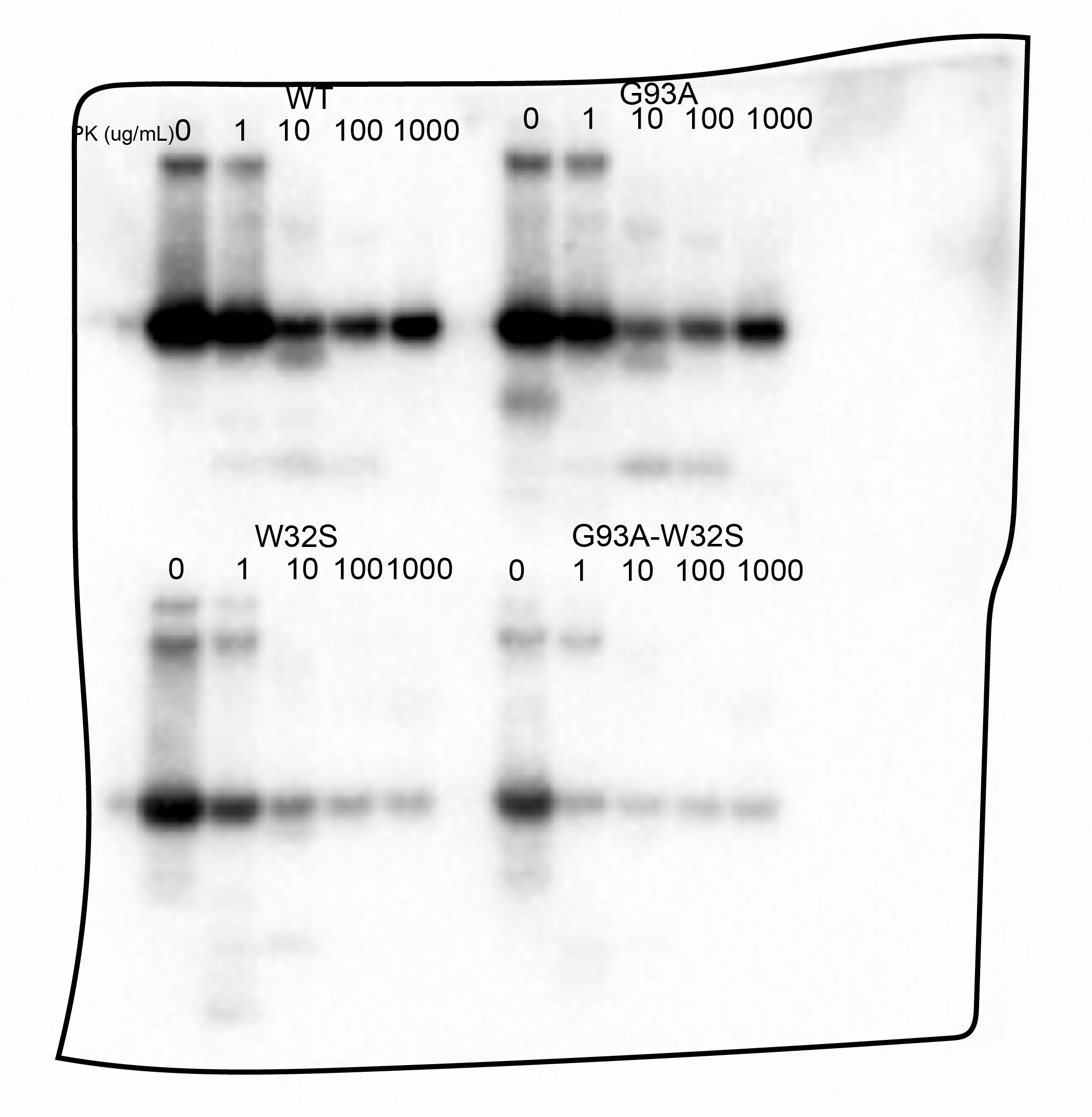


**Supplementary Figure 4. Uncropped blots from supplementary figure 2. Apo-SOD1 digested by Proteinase K.** The black outline denotes the edge of the blot membrane.


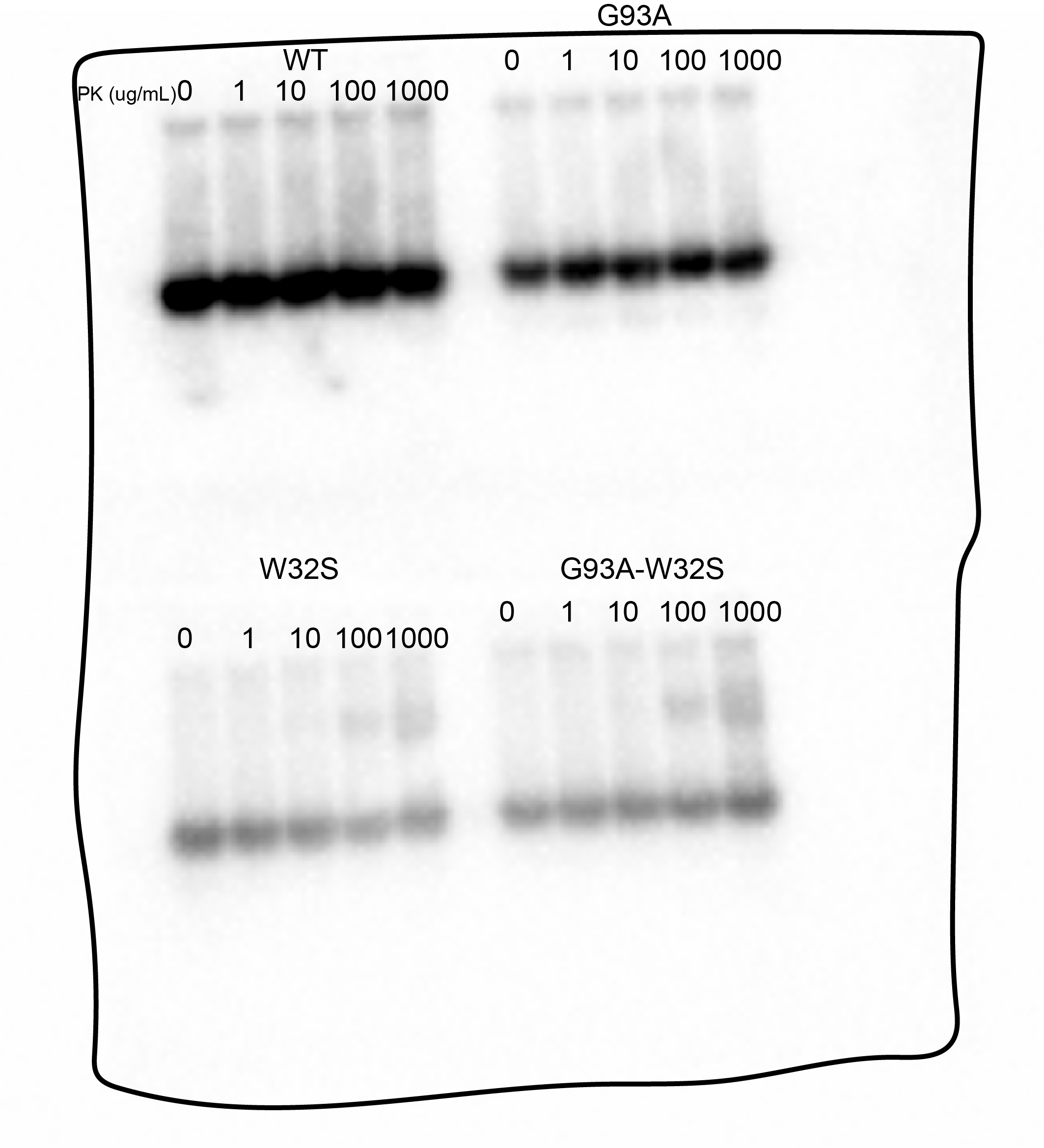


**Supplementary Figure 5. Uncropped blots from supplementary figure 2.** Holo-SOD1 digested by Proteinase K. The black outline denotes the edge of the blot membrane.

**
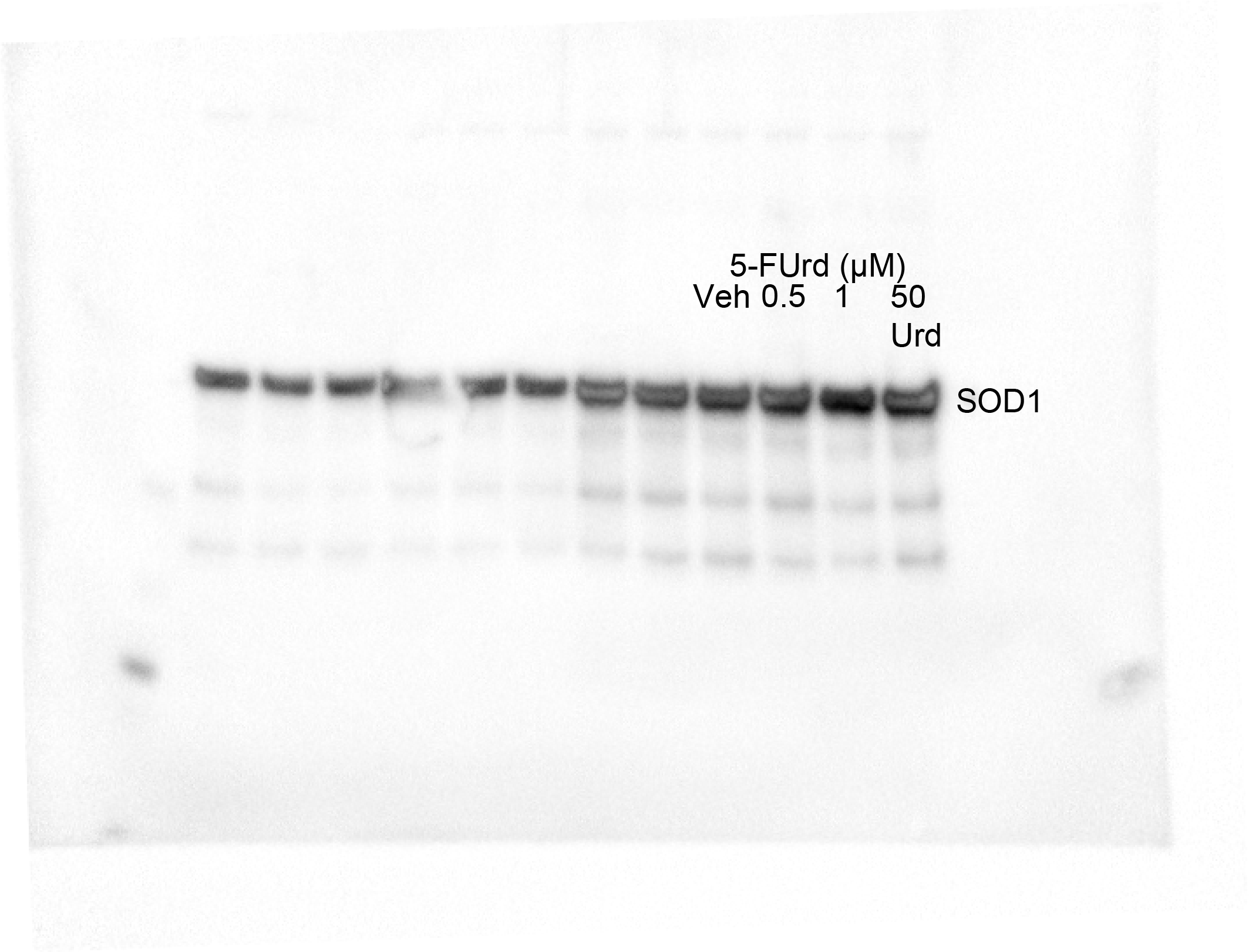
**

**Supplementary Figure 6. Uncropped blot from Figure 5 panel C.** SOD1-GFP detected in cells treated with 5-FUrd.

**
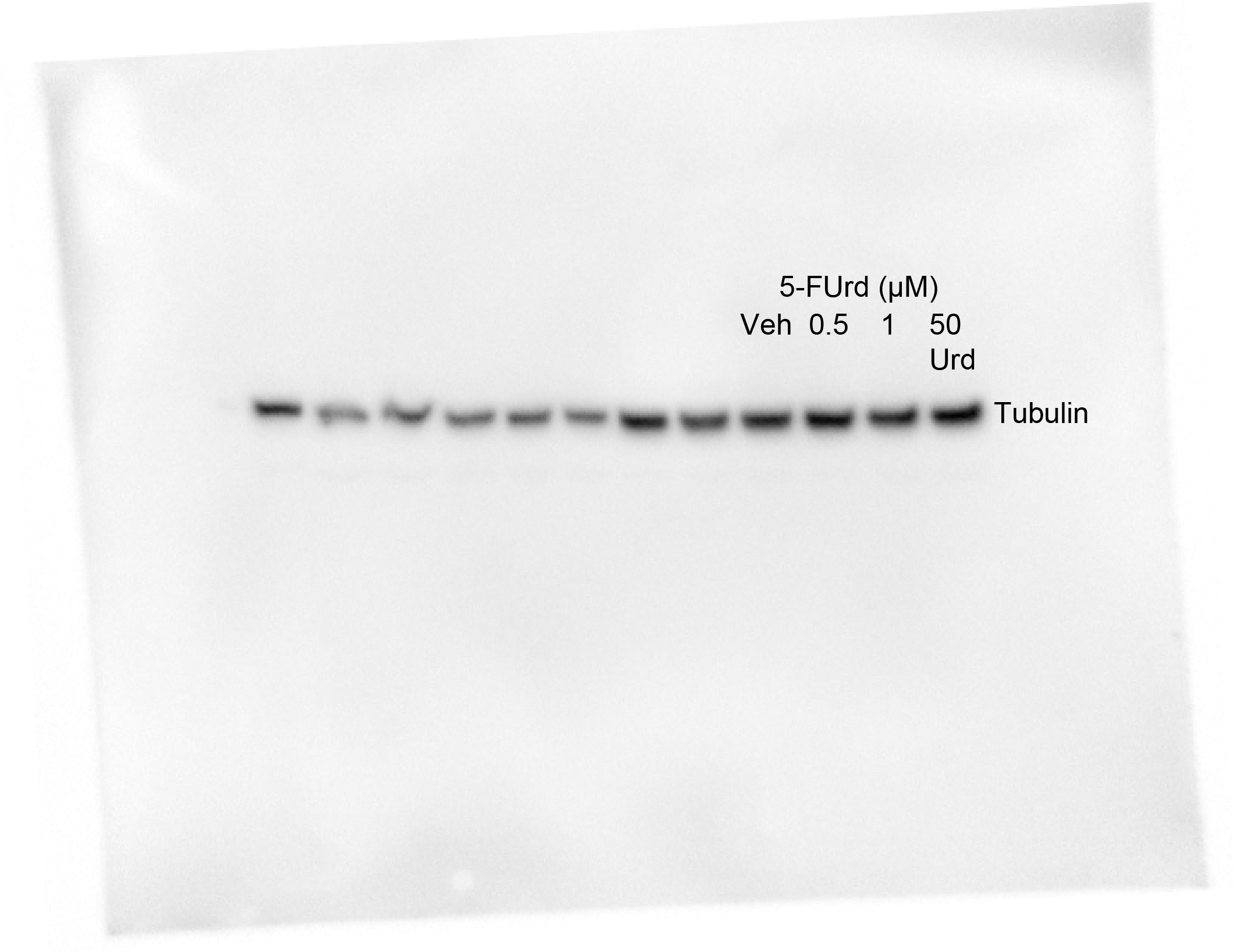
**

**Supplementary Figure 7. Uncropped blot from Figure 5 panel C.** Tubulin from SOD1-GFP cells treated with 5-FUrd.

**
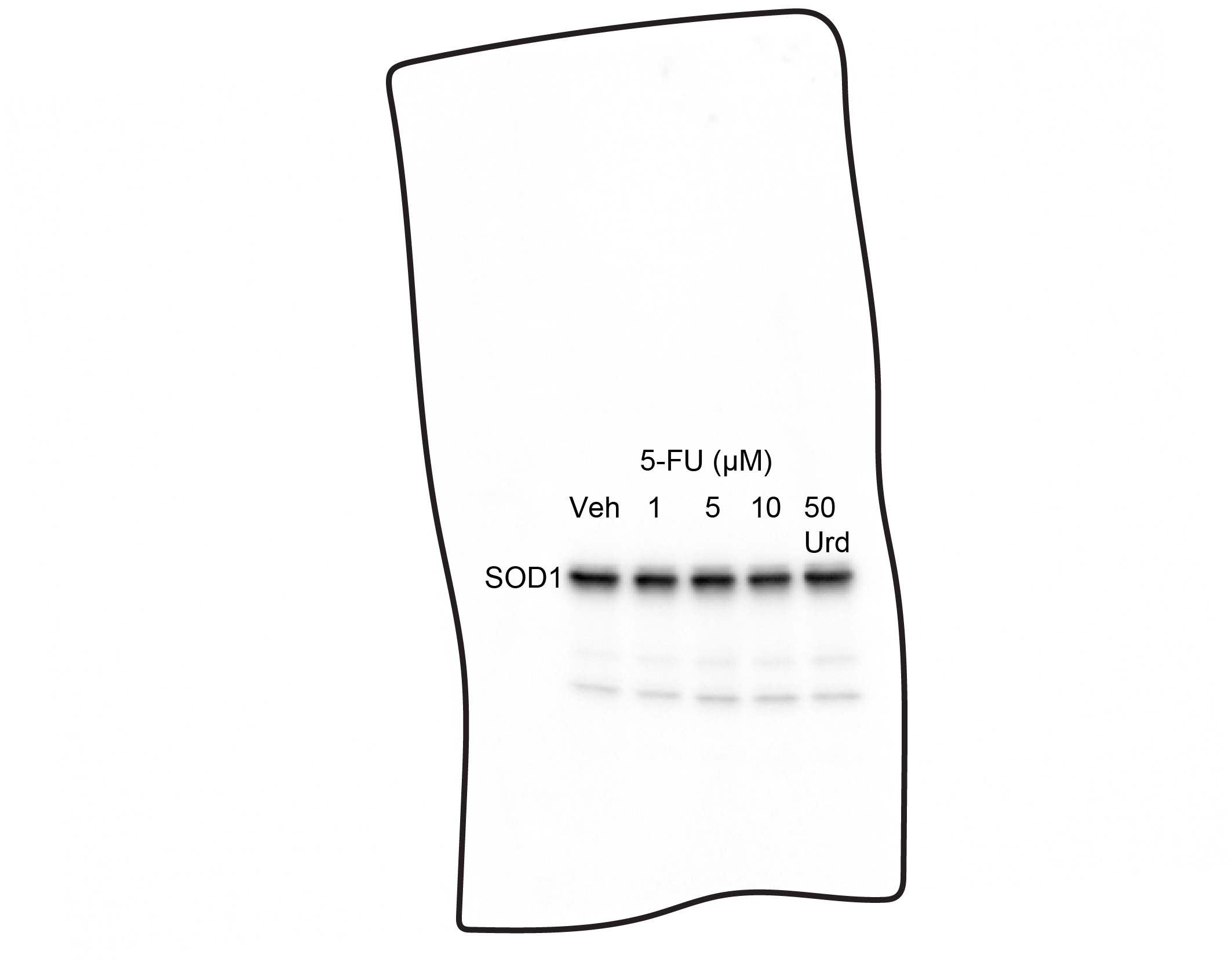
**

**Supplementary Figure 8. Uncropped blot from Figure 5 panel C.** SOD1-GFP from cells treated with 5-FU. Black outline denotes the edge of the blot membrane.


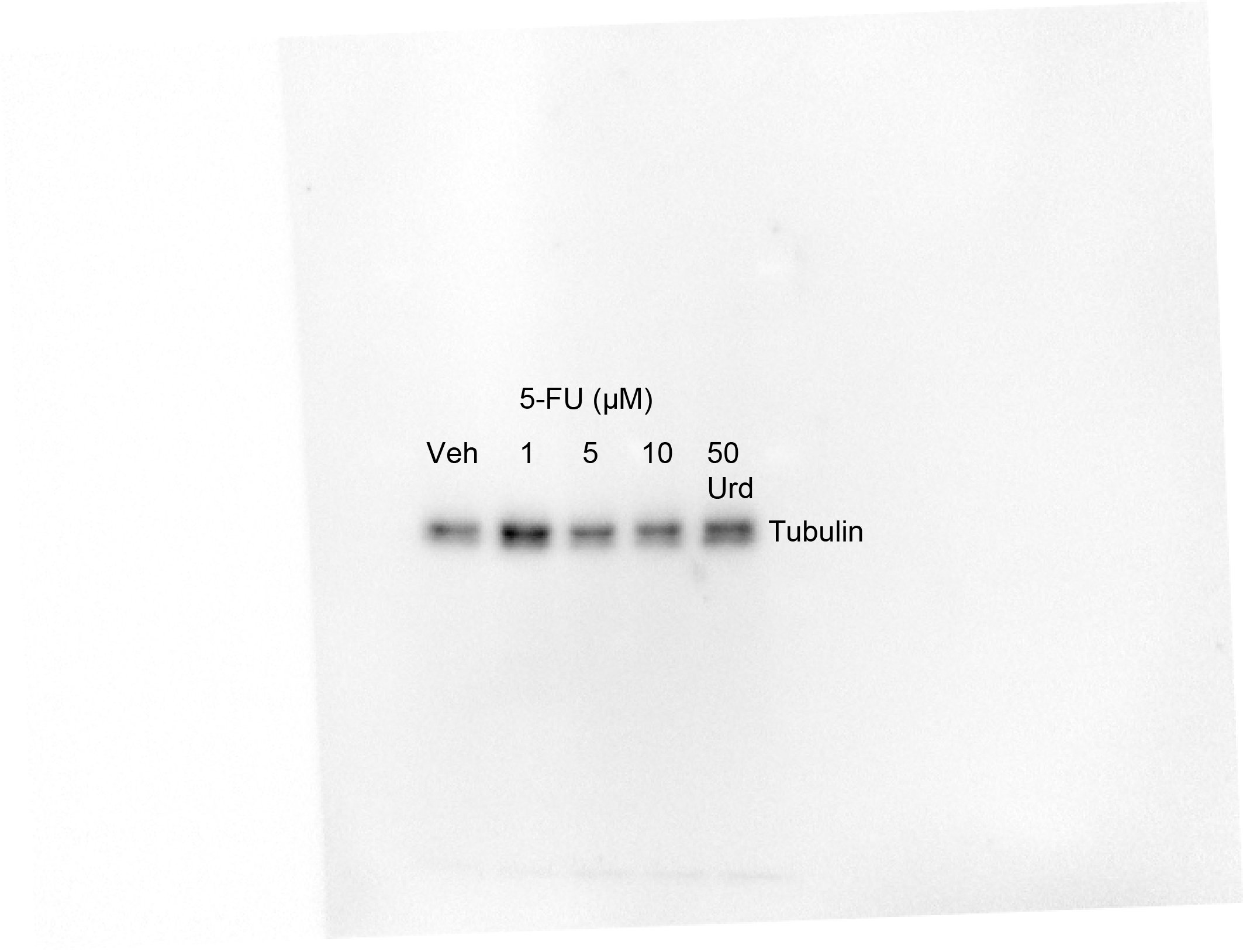


**Supplementary Figure 9. Uncropped blot from Figure 5 panel C.** Tubulin from cells transfected with SOD1-GFP treated with 5-FU.


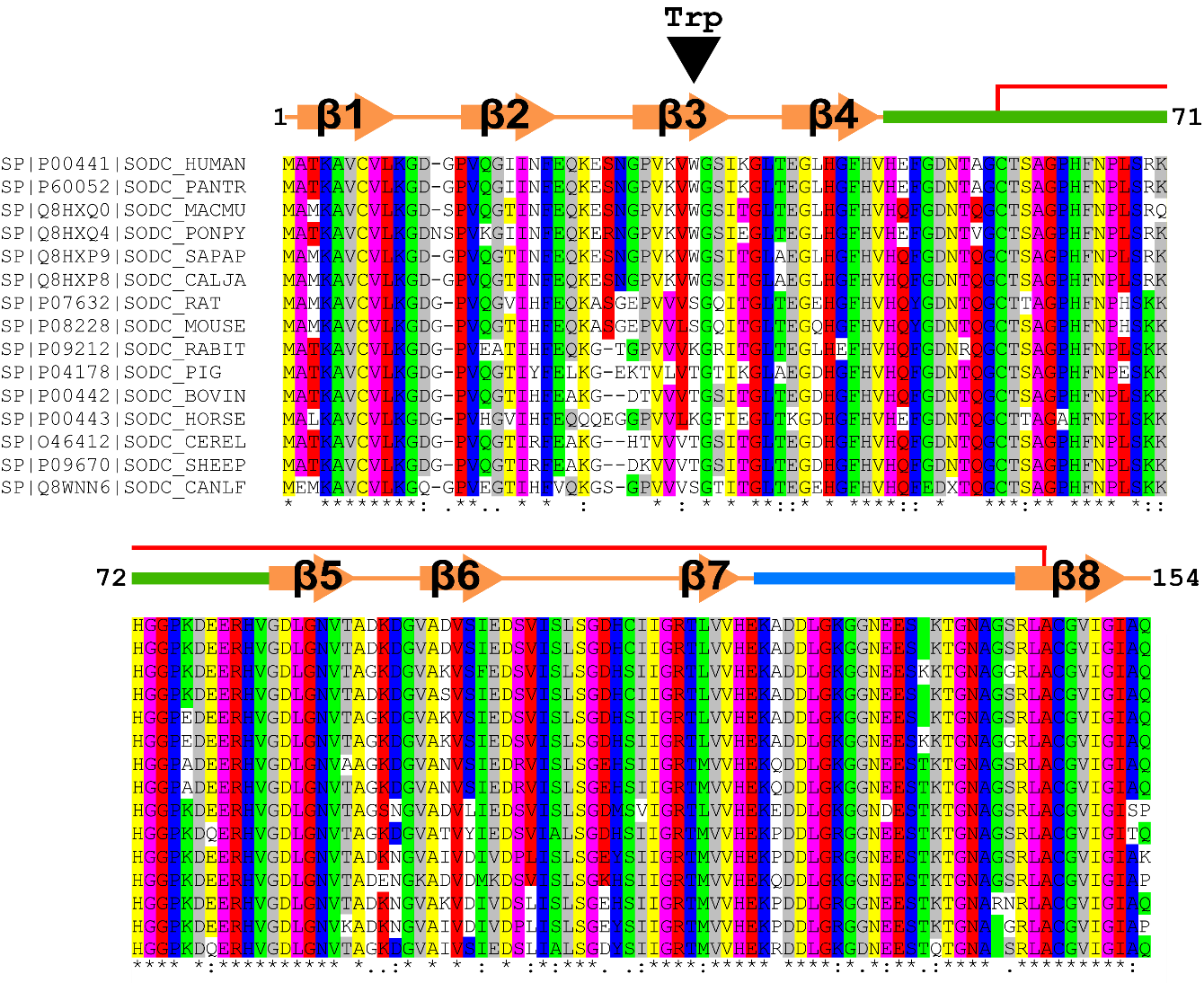


**Supplementary Figure 10. Multiple sequence alignment of the SOD1 protein sequence.** Reviewed accessions from uniprot were aligned to determine conservation of amino acids. Tryptophan-32 (black arrow) is conserved in primates but not present in other analysed mammals.

**Movie S1.** Time-lapse fluorescent microscopy of HEK293FTcells transfected with either G85R-W32-GFP or G85R-S32-GFP.

**Movie S2.** Time-lapse fluorescent microscopy of HEK293FT cells co-transfected with G85R-W32-GFP reporter protein and either empty vector control, G85R-W32, or G85R-S32 as aggregation inducers.

**Movie S3.** Time-lapse fluorescent microscopy of HEK293FT cells co-transfected with G85R-W32-GFP reporter and G85R-W32 aggregation inducer with drug treatments including vehicle control, 50 µM uracil, 0.5 µM 5-FUrd, and 5 µM 5-FU.

**Materials and Methods**

***Plasmids used for recombinant protein production***

Expression vectors encoding SOD1-WT and SOD1-G93A for bacterial expression were a gift from Professor Mikael Oliveberg (Stockholm University, Sweden). Plasmids encoding SOD1-V148G, and S32 constructs (SOD1-S32, SOD1-G93A-W32S, SOD1-V148G-W32S) were designed in-house and generated by Genscript (USA).

***Plasmids for mammalian expression***

Plasmids for the expression of SOD1 in mammalian cells included pEGFP-N1 encoding SOD1-WT, SOD1-A4V, SOD1-G85R, SOD1-G93A, and SOD1-V148G (as well as their
S32 counterparts). The EGFP in pEGFP-N1 was replaced by tdTomato encoding SOD1-A4V, SOD1-G85R, SOD1-G93A, and SOD1-V148G as previously described (Farrawell et al., 2015). Plasmid containing SOD1-G85R-AcGFP reporter protein was a gift from Professor Elizabeth Fisher (University College London, UK) (Stevens et al., 2010). The plasmid for the expression of the tdTomatoCL1 construct was obtained by cloning the CL1 sequence (ACKNWFSSLSHFVIHL) into pcDNA3.1(+)tdTomato. The plasmid for the expression of XBP1-tdTomato was generated by replacing the venusYFP in the pCAX-F-XBP1-venus, a kind gift from Dr. Masayuki Miura (Tokyo University, Japan) with tdTomato.

***Expression and purification of recombinant SOD1***

Protein expression and purification were performed according to previous methods (Lindberg et al., 2002). Briefly, SOD1 expression vectors were transformed into competent BL21(DE3) *E. coli* via heat-shock. Expression cultures in 2 × LB were grown until an OD_600_ of 0.6, at which point ZnSO_4_ and CuSO_4_ were added to 200 µM and 3 mM respectively, and expression was induced by IPTG (0.5 mM) followed by overnight incubation at 18°C with orbital shaking (200 rpm). Following overnight incubation, cells were collected via centrifugation (6200 × *g*, 10 min), resuspended in 50 mM Tris-base (pH 7.4), and lysed using an Emulsiflex-C5 high pressure homogeniser (Avestin, Canada) using two passes. Homogenized material was cleared via centrifugation (40 000 × *g*, 20 min) and the supernatant was subject to heat denaturing (65°C for 30 min) followed by centrifugation (40 000 × *g*, 20 min). Solid ammonium sulphate was added to the supernatant to a concentration of 60% (w/v) at 4°C with mild stirring, and the sample was incubated for 2 h, following which precipitated protein was cleared via centrifugation (40 000 × *g*, 20 min). Ammonium sulphate was added to the supernatant to a final concentration of 90% with stirring at 4°C, and the sample was incubated overnight. Following overnight incubation, samples were cleared by centrifugation (40 000 × *g,* 20 min) and the pellet was carefully rinsed with distilled H_2_Oand resuspended in 50 mM Tris 150 mM NaCl (pH 7.4). Samples were loaded onto a gel filtration column (Hiload 16/60 Superdex 75 PG, GE, USA) equilibrated in 50 mM Tris 500 mM NaCl (pH 7.4) and eluted at a flow rate of 1 mL/min. Fractions containing SOD1 (determined by SDS-PAGE) were pooled and dialysed into 20 mM Tris (pH 8) using SnakeSkin™ dialysis tubing (Thermofisher, USA) with three buffer changes. Dialysed sample was then loaded onto an anion exchange column (HiTrap DEAE, GE USA) equilibrated in 20 mM Tris (pH 8) buffer and was eluted across a 0 – 250 mM NaCl gradient. Fractions containing pure SOD1 (determined by SDS-PAGE) were pooled and concentrated using centrifugal concentrators (Vivaspin 20, 10 000 MWCO, GE USA). Concentrated samples were flash frozen in liquid nitrogen and stored at ‑20 °C until use.

***Transmission electron microscopy***

A volume of 2 µL of aggregated SOD1 from ThT binding assays was loaded onto a carbon coated 400 mesh Nickel TEM grid (ProSciTech, Australia). Loaded samples were then diluted with 10 µL of 0.22 µm filtered milli-Q H_2_O and left to stand for 1 min. Grids were dried with fine fibre blotting paper by dabbing the side gently. The addition of milli-Q H_2_O and drying was repeated three times, after which 10 µL of 2% uranyl acetate (w/v) was added and left to stand for 1 min. Uranyl acetate was dried off the grid gently with blotting paper, and the grid was left to air dry, upside down, for 5 min before being stored. Grids were stored at room temperature in a sealed plastic container. Prepared grids were imaged at the Australian Institute of Innovative Materials using a JEOL JEM-2011 transmission electron microscope (JEOL, Japan). Images were processed using Image J software.

***Differential Scanning Fluorimetry***

SOD1 was demetallated according to previous methods (McCord and Fridovich, 1969). Following removal of metals, apo-SOD1 was extensively dialysed into 10 mM HEPES 150 mM NaCl (pH 7.4), flash frozen and stored at -20 °C. Samples were defrosted on ice and plated into a 96-well fast-PCR plate (Thermofisher, USA) at a concentration of 20 µM with 20X SYPRO Orange (ThermoFisher, USA). A QuantStudio-6 Real-Time PCR machine (Thermofisher, USA) was used to measure dye fluorescence with a FAM filter-set. Plates were heated at a rate of 0.016 °C/s until a final temperature of 95 °C was reached. An in-house python script was written to calculate the melting temperature (T_m_) from the first derivative of the raw data.

***Native Mass Spectrometry***

Purified SOD1 was destabilized via incubation in 20 mM DTT 5 mM EDTA in 1 × PBS (pH 7.4) at 37°C for 2 h, following which samples were buffer exchanged into 200 mM ammonium acetate using gel-filtration chromatography (Superdex 75 10/300 GL, GE USA). Fractions off the column were immediately placed on ice, pooled, and diluted to a final concentration of 10 µM SOD1 monomer (measured via BCA assay). Mass spectrometry analysis was performed using a SYNAPT G1 HDMS (Waters, UK) with parameters set according to previous work (McAlary et al., 2013) (Table 1). Briefly, SOD1 samples at 10 μM in 200 mM NH_4_OAc were loaded into gold-coated borosilicate capillaries (made in-house) and subjected to nano-electrospray ionization. Results are representative of 3 separate unfolding experiments. All spectra were externally calibrated using 10 mg/ml caesium-iodide in 50% n-propanol, and were processed using Masslynx 4.1. For determination of the abundances of the observed conformations the area under the peak for each charge state was determined and the values plotted as a function of charge state. Following this, MATLAB R2014b (Version 8.4) was used to fit Gaussians to the plots and area under the peak was determined. Instrument parameters can be found in table 1.

Supplementary Table 2. System parameters for SYNAPT G1 HDMS.

| ***Parameter*** | ***Experimental Setting*** |
| --- | --- |
| Capillary voltage | 1.52 (kV) |
| Sampling Cone | 80 V |
| Extraction Cone | 4.0 V |
| Source Temperature | 25 °C |
| Low Mass Resolution | 4.7 |
| High Mass Resolution | 15.0 |
| Trap Collision Energy | 6 V |
| Transfer Collision Energy | 4 V |
| Trap Gas Flow | 2.9 mL/min (Nitrogen) |
| Source Gas Flow | 1 mL/min (Argon) |
| Detector | 1800 V |
| Backing Pressure | 4.0e^0^ mbar |
| Trap Pressure | 1.21e^-1^ mbar |
| Time-of-Flight Pressure | 8.41e^-2^ mbar |

***Proteolytic analysis***

25 µg of purified recombinant apo- or holo-WT, W32S, G93A, and G93A-W32S in 1 × PBS were treated with proteinase K at 0, 1, and 10 µg/mL for 1 h at 37 °C. Reactions were terminated by placing digests on ice and the addition of proteinase K specific inhibitor phenylmethylsulfonyl fluoride to a final concentration of 5 mM. Samples were run on NuPAGE 4-12% gels (Thermofisher, USA) and blotted with SOD100 antibody (Enzo Biochem Inc, USA).

***Tissue culture***

Mouse neuroblastoma/motor neuron hybrid cells (NSC-34) (Cashman et al., 1992) were cultured in Dulbecco's modified Eagles medium-F12 (DMEM-F12) (Invitrogen, USA), supplemented with 10% (v/v) heat inactivated fetal bovine serum (FBS) (Bovogen Biologicals, Australia). In order to passage and plate cells, they were washed once with DMEM-F12 and treated with 0.25% trypsin, 0.02% EDTA (Invitrogen, USA) to lift off the adherent cells. The cells were pelleted via centrifugation (500 × *g*, 5 min) and resuspended in pre-warmed DMEM-F12, supplemented with 10% FBS. Following washing, plates and chamber-slides were seeded at a confluency of 40% and cultured at 37°C in a humidified incubator with 5% atmospheric CO_2_ for 24 h prior to transfection (~70-80% confluent). NSC-34 cells were transfected, 24 h post-plating into either multi-well plates or 8-well chamber-slides (Ibidi, Germany), using Lipofectamine 3000 (Invitrogen, USA) according to the manufacturer's instructions.

Human embryonic kidney cells (HEK293FT; ATCC) were cultured in complete Dulbecco’s Modified Eagle Medium (DMEM) containing 10% FBS, 10 U/mL penicillin, 10 U/mL streptomycin and 2 mM L-glutamine (Thermo Fisher, USA). Cell were plated and transiently transfected at 40-60% confluency with SOD1 plasmid DNA using Lipofectamine LTX (Thermo Fisher, USA), according to manufacturer’s instructions.

***Immunoblotting drug treated cells***

HEK293FT cells were transfected with SOD1-G85R-AcGFP and treated with indicated concentrations of compounds approximately 6 h post-transfection. 48 h post-transfection, cells were harvested in 1 × PBS and lysed (1% Triton X-100 in PBS+ cOmplete EDTA-free protease inhibitor tablet). Lysate was run on NuPAGE 4-12% gels before being immunoblotted. SOD1-GFP was detected with anti-GFP antibody (FL sc-8334 – Santa Cruz Biothecnology, USA). Alpha-tubulin was detected with anti-alpha tubulin (66031-1-Ig – Proteintech, USA).

***Fluorescent imaging***

To assess the toxicity of SOD1-EGFP constructs in NSC-34 cells over a time course, an IncuCyte® automated fluorescent microscope (Essen BioScience, USA) was used according to previous work (McAlary et al., 2016). Briefly, NSC-34 cells were plated into 12-wells plates at a confluency of 60% and were transfected 24 h post-plating. Cells were dissociated 24 h post-transfection with trypsin and plated into 96-well plates at a confluency of 20% in phenol-red-free DMEM-F12 supplemented with 10% FBS. At least 3 images were acquired per-well using a 10× objective at 2 h time points for 68 h in both phase and green channels with the green channel exposure time at 400 ms. The processing definition generated to analyse the images utilized top-hat back ground subtraction [radius = 100 μm, threshold = 0.5 general calibration units (GCU)], edge-splitting (edge sensitivity = 0), filters (minimum area = 175 μm2, minimum mean intensity = 1.2). The equation set 1in the appendices below describe data analysis and presentation.

First, the number of GFP positive cells at each time point (GFP_tx_) for each transfection was normalized to the intial value (GFP_t0_) determined in the first scan after plating.

$$\frac{{GFP}_{tx}}{{GFP}_{t0}}={Normalised GFP}_{tx}$$

Then, the normalized values of the SOD1 mutants at each time point (Normalised GFPtx) were divided by the normalized SOD1WT data at the same time points to determine the proportion of GFP positive cells relative to SOD1WT.

$$\frac{{Normalised GFP}_{tx}}{{SOD1WT-GFP}_{tx}}=SOD1 mutant GFP relative to SOD1 WT$$

Imaging of transiently transfected NSC-34 cells was performed using 8-well microscope chambers 48 h post-transfection using an inverted TCS SP5 laser-scanning confocal microscope (Leica, Germany). Cells were imaged live in 5% atmospheric CO_2_ at 37°C using a HCX PL APO 63.0× 1.30NA water immersion objective with the argon laser power at 20%, with the DPSS 561 nm laser being turned on for tdTomato transfections. SOD1-EGFP constructs were excited with 10% laser power at 488 nm and emission was read from 507-550 nm, whereas SOD1-tdTomato constructs were excited with 7% laser power at 561 nm and emission was read from 581-700 nm. Both channels were read with PMT detectors. Bleed through of GFP signal into the tdTomato emission window was minimised using frame by frame sequential scanning. Both EGFP and tdTomato channels were gain adjusted in over-glow mode to avoid saturation of the images acquired. Images were acquired at 512 × 512 pixel resolution using a laser scan speed of 400 Hz, and analysed using Image J. Determination of the percentage of cell containing inclusions in NSC-34 cells was performed by acquiring z-stacks of at least 5 fields of view encompassing at least 100 cells per replicate. Z-stack images were projected using maximum intensity and the number of cells containing inclusions was counted by eye.

For antibody staining, cells were plated into glass coverslips prior to transfection with the indicated construct as described above and collected 48 h post-transfection. Cells were fixed using 4% paraformaldehyde and stained with misfolding specific antibody 3H1, and pan-SOD1 antibody SOD100 (Enzo Life Sciences, USA) as previously described (Pokrishevsky et al., 2016).

***Image Analysis***

This section is related to figure 4. Since intracellular SOD1 aggregates are noted to be porous ([1-3](#_ENREF_1)), corrected aggregate fluorescence intensity (*Aggregated* *I_C_*) was determined by subtracting the diffuse mean pixel intensity (*Diffuse* *I_P_*) from aggregated mean pixel intensity (*Aggregated* *I_P_*).

| 1. | ${(Aggregated I}_{P})-\left( Diffuse I_{P} \right)=Corrected Aggregated I_{P} (Aggregated I_{C})$ |
| --- | --- |

Total aggregated signal (*S_A_*) was determined by multiplying the corrected aggregated fluorescence intensity (*Aggregated* *I_C_*) by the aggregated pixel area (*Aggregated* *A_P_*).

| 2. | ${(Aggregated I}_{C})\times{(Aggregated A}_{P})=Total Aggregated Signal (S_{A})$ |
| --- | --- |

Total diffuse signal (*S_D_*) was determined by multiplying the mean diffuse fluorescence intensity (*Diffuse* *I_P_*) by the diffuse pixel area (*Diffuse* *A_P_*).

| 3. | $\left( Diffuse I_{P} \right)\times\left( Diffuse A_{P} \right)=Total Diffuse Signal (S_{D})$ |
| --- | --- |

Total cell fluorescence signal (*S_C_*) was determined by the addition of total diffuse signal (*S_D_*) and total aggregated signal (*S_A_*).

| 4. | $S_{D} + S_{A}=Total Cell Signal (S_{C})$ |
| --- | --- |

The proportion of aggregated fluorescent signal (*P_A_*) was determined by dividing the total aggregated signal (*S_A_*) by total cell fluorescence signal (*S_C_*).

| 5. | $\frac{S_{A}}{S_{C}}=Proportion Aggregated \left( P_{A} \right)$ |
| --- | --- |

The proportion of aggregated GFP (*GFP P_A_*) was then divided by the proportion of aggregated tdTomato (*tdTomato P_A_*) to determine the ratio of GFP to tdTomato fluorescence signal that was contributing to the intracellular inclusions.

| 6. | $\frac{{(GFP P}_{A})}{(tdTomato P_{A})}=Ratio of GFP to tdTomato aggregated$ |
| --- | --- |

***Flow cytometry***

To assay ubiquitin proteasome dysfunction, NSC-34 cells were co-transfected with either GFP, WT-GFP, G85R-GFP, or G85R-S32-GFP, and tdTomato-CL1. Positive control of 20 µM MG132 was used to confirm UPS impairment. Co-transfected cells were harvested 48 h post-transfection with trypsin and resuspended into 1× PBS. The fluorescence intensity of tdTomato in co-transfected cells was measured using a BD LSRFortessa X-20 Cell Analyzer (BD Biosciences, USA). Analysis was performed using FlowJo version 10 (FlowJo LLC, USA) and compensation was performed using the in-built compensation wizard. Assays of ER-stress based on XBP1 splicing were performed in a similar fashion to the CL1 degron experiments, using the XBP1-tdTomato construct and 2.75 µM thapsigargin as a positive control.

**Supplementary References**

1. Farawell N*, et al.* (2015) Distinct Partitioning of ALS Associated TDP-43, FUS and SOD1 mutants into cellular inclusions. *Scientific Reports* 5:13416.

2. Matsumoto G, Kim S, & Morimoto R (2006) Huntingtin and Mutant SOD1 form Aggregate Structures with Distinct Molecular Properties in Human Cells. *Journal of Biological Chemistry* 281(7):4477-4485.

3. Matsumoto G, Stojanovic A, Holmberg C, Kim S, & Morimoto R (2005) Structural Properties and Neuronal Toxicity of Amyotrophic Lateral Sclerosis-Associated Cu/Zn Superoxide Dismutase 1 Aggregates. *The Journal of Cell Biology* 171(1):75-85.
